# Diagnostic utility of prostate health index density prior to MRI-ultrasound fusion targeted biopsy

**DOI:** 10.37349/etat.2024.00269

**Published:** 2024-09-13

**Authors:** Benjamin H. Press, Soum D. Lokeshwar, Lindsey Webb, Ghazal Khajir, Shayan Smani, Olamide Olawoyin, Mursal Gardezi, Syed N. Rahman, Michael S. Leapman, Preston C. Sprenkle

**Affiliations:** Queen Mary University of London (QMUL), UK; IRCCS Istituto Romagnolo per lo Studio dei Tumori (IRST) “Dino Amadori”, Italy; Department of Urology, Yale School of Medicine, New Haven, CT 06510, USA

**Keywords:** Prostatic neoplasms, prostate-specific antigen, protein isoforms, MRI, image-guided biopsies

## Abstract

**Aim:**

Prostate biopsy can be prone to complications and thus should be avoided when unnecessary. Although the combination of magnetic resonance imaging (MRI), the prostate health index (PHI), and PHI density (PHID) has been shown to improve detection of clinically significant prostate cancer (csPCa), there is limited information available assessing its clinical utility. We sought to determine whether using PHID could enhance the detection of PCa on MRI ultrasound fusion-targeted biopsy (MRF-TB) compared to other biomarker cutoffs.

**Methods:**

Between June 2015 and December 2020, 302 men obtained PHI testing before MRF-TB at a single institution. We used descriptive statistics, multivariable logistic regression, and receiver operating characteristic curves to determine the predictive accuracy of PHID and PHI to detect ≥ Gleason grade group (GGG) 2 PCa and identify cutoff values.

**Results:**

Any cancer grade was identified in 75.5% of patients and ≥ GGG2 PCa was identified in 45% of patients. The median PHID was 1.05 [interquartile range (IQR) 0.59–1.64]. A PHID cutoff of 0.91 had a higher discriminatory ability to predict ≥ GGG2 PCa compared to PHI > 27, PHI > 36, and prostate specific-antigen (PSA) density > 0.15 (AUC: 0.707 vs. 0.549 vs. 0.620 vs. 0.601), particularly in men with Prostate Imaging Reporting and Data System (PI-RADS) 1–2 lesions on MRI (AUC: 0.817 vs. 0.563 vs. 0.621 vs. 0.678). At this cutoff, 35.0% of all the original biopsies could be safely avoided (PHID < 0.91 and no ≥ GGG2 PCa) and csPCa would be missed in 9.67% of patients who would have been biopsied. In patients with PI-RADS 1–2 lesions using a PHID cutoff of 0.91, 56.8% of biopsies could be safely avoided while missing 0 csPCa.

**Conclusions:**

These findings suggest that a PHID cutoff of 0.91 improves the selection of patients with elevated prostate-specific antigen who are referred for prostate biopsy, and potentially in patients with PI-RADS 1–2 lesions.

## Introduction

Conventional prostate specific-antigen (PSA) based screenings for prostate cancer (PCa) have favored lower thresholds with greater sensitivity for cancer detection [1]. However, the majority of men with a PSA 4–10 ng/mL do not have PCa with an incidence of only 20–30% [2, 3]. Thus, one sustained challenge in the early detection of PCa has been balancing the identification of potentially lethal cancer with avoiding over-detection of indolent disease. One key factor contributing to the controversy of screening and diagnosis of PCa is the subsequent steps that follow an elevated PSA in clinical practice. Over 1 million men undergo prostate biopsy in both the United States and Europe each year [4]. Regardless of approach, the procedure is associated with risks of complications including rectal bleeding, hematuria, hematospermia, urinary retention urinary tract infections, and sepsis [4]. Even with the now-widespread use of magnetic resonance imaging (MRI) to evaluate for prostate lesions suspicious for clinically significant PCa (csPCa), often defined as histopathological Gleason grade group (GGG) ≥ 2 [5], a reliable subset of men without concerning lesions on MRI still have occult csPCa [6]. Additionally, a significant barrier to biopsy compliance for patients with longer follow-ups on surveillance is the requirement for serial prostate biopsy [7, 8]. This may lead to treatment of insignificant disease or untreated disease progression due to patient refusal to complete recommended biopsies. Identifying which men are candidates to safely avoid unnecessary biopsies may be able to improve compliance with necessary biopsies. Therefore, the need for increasingly personalized risk stratification exists.

In 2012, the FDA approved the prostate health index (PHI) for men with PSA 4–10 ng/mL to predict the probability of PCa before biopsy [9]. PHI is a formula that combines three forms of PSA into a single score: ([–2]proPSA/free PSA) × √PSA. More recently, PHI density (PHID), a parameter reflecting the ratio of PHI score to prostate volume, has been shown to improve detection of csPCa [10]. Although the combination of MRI and PHI and PHID has been shown to improve detection of csPCa [11], there is still limited information available to guide clinical management. MRI has been shown to be a tool to identify those who do not require biopsy. MRI-targeted biopsy was able to detect both csPCA and less indolent disease, demonstrating its potential as a screening tool [12]. MRI reads contain prostate volume estimations, making PHID an easy number to calculate. We sought to determine whether PHID could further optimize the detection of PCa on MRI ultrasound fusion-targeted biopsy (MRF-TB). Additionally, we sought to further compare the utility of a PHID cutoff compared to cutoffs of other commonly employed biomarkers for the detection of csPCa.

## Materials and methods

### Study design and patient selection

Data was analyzed from a retrospectively collected IRB-approved database. We identified patients who obtained PHI testing prior to MRF-TB at our institution. Findings on prostate biopsy, including Gleason grade (GG), Prostate Imaging Reporting and Data System (PI-RADS), and PHI were recorded. Obtaining PHI was recommended, but not required, for patients before biopsy. Different cutoff points proposed in the literature for PHI and PSA density (PSAD) were evaluated for cancer detection rates: PHI > 27, PHI > 36, and PSAD > 0.15 [13, 14]. PHID was calculated by dividing the PHI by the MRI-measured prostate volume and, using receiver operating characteristic (ROC) curves, a PHID cutoff was determined.

### Multiparametric MRI

MRI was conducted using a 3-tesla system and pelvic phased-array coil, incorporating multiplanar T2-weighted images, axial diffusion-weighted imaging with b-values of 50 s/mm^2^ and 1,000 s/mm^2^, and dynamic contrast-enhanced imaging received after the administration of gadolinium chelate. Skilled genitourinary radiologists evaluated multiparametric MRI (mpMRI) studies using the PI-RADS v2, categorizing findings on a scale from 1 to 5.

### MRI-ultrasound fusion-targeted biopsy

MRF-TB was conducted utilizing an Artemis prostate biopsy system alongside ProFuse (Eigen, Grass Valley, CA, USA) software for MRI segmentation, three-dimensional biopsy planning, and co-registration of MRI and US images. Suspicious lesions identified on T2-weighted MRI sequences were transferred to the Artemis system. Computer-assisted co-registration of prostatic TRUS and segmented MRI images was achieved through a combination of manual rigid translation and automated elastic deformation. Patients were positioned in the left lateral decubitus position for transrectal biopsy. The biopsy procedure began with five targeted biopsy cores corresponding to each suspicious lesion identified on mpMRI, followed by an additional 12 software-populated, spatially distributed cores. Importantly, the sites selected for 12-core sampling were determined by the Artemis device, without input from the operating surgeon.

### Statistical analysis

Categorical variables were reported as *n* (%) while continuous variables were reported with median and interquartile range (IQR). Statistical analysis was performed using SPSS version 27 (IBM SPSS Statistics, Armonk, NY, USA). McNemar’s test was used for statistical analysis of proportions. The Kruskal-Wallis test was used for statistical analysis of ordinal and continuous, non-normally distributed variables. Multivariable logistic regression and ROC curves were used to assess the predictive capacity of PHI and PHID cutoffs to detect ≥ GGG2 PCa. The cutoff value for PHID to detect ≥ GGG2 PCa was determined from the Youden Index on ROC curves.

## Results

Between June 2015 and December 2020, a total of 302 men underwent prostate biopsy after measurement of PHI. Baseline patient characteristics can be seen in Table 1. Two hundred sixty-six of these patients received their first biopsy or were placed on active surveillance (AS). The median age of our cohort was 66.9 years with a median PSA of 6.35 ng/mL and a median PSA density of 0.124 ng/mL^2^. Median PHI was 50.5, with 91.7% above a value of 27 and 77.4% above a value of 36. The median PHID was 1.05 (IQR 0.59–1.64). The overall cancer detection rate was 75.5% with a 45.0% rate of detection of ≥ GGG2 PCa. Of the 302 men biopsied, 112 were on AS and 154 underwent their first biopsy. The mean PHI for patients with a maximum PI-RADs of 1, 2, 3, 4, and 5 was 53.877 (IQR 35.775–69.75), 50.322 (IQR 29.85–65.975), 49.003 (IQR 32.3–60.3), 54.06 (IQR 39.15–64.375), and 70.505 (IQR 45.3–88.4), respectively. There was not a statistically significant difference in PHI between patients with maximum PI-RADs scores of 1, 2, 3, and 4 (*P* = 0.363), however, with the inclusion of patients with a PI-RADs 5 lesion, PHI was significantly different (*P* = 0.002).

**Table 1:** Baseline patient characteristics

**Variable**	**Total cohort** (*N* = 302)^*^	**Active surveillance** **(*N* = 112)**	**First biopsy** **(*N* = 154)**
Age (years), median (IQR)	66.91 (62.25–71.54)	66.52 (62.36–70.89)	66.98 (62.13–71.80)
PSA (ng/mL), median (IQR)	6.35 (4.80–8.75)	6.73 (4.78–9.39)	5.91 (4.74–7.76)
PSA density (ng/mL^2^), median (IQR)	0.124 (0.085–0.184)	0.124 (0.78–0.20)	0.127 (0.089–0.177)
PHI, median (IQR)	50.5 (37.90–68.20)	49.1 (38.3–65.8)	53 (39.68–68.7)
> 27	276 (91.7%)	102 (91.1%)	143 (92.9%)
> 36	233 (77.4%)	88 (78.6%)	120 (77.9%)
PHID, median (IQR)	1.05 (0.59–1.64)	1.05 (0.55–1.60)	1.15 (0.70–1.72)
PI-RADS, *N* (%)			
1	31 (10.3%)	14 (12.5%)	11 (7.1%)
2	50 (16.6%)	18 (16.1%)	22 (14.3%)
3	31 (10.3%)	9 (8.0%)	18 (11.7%)
4	134 (44.5%)	48 (42.9%)	74 (48.1%)
5	55 (18.3%)	22 (19.6%)	29 (18.8%)
Prostate cancer detection rate, *N* (%)	228 (75.5%)	93 (83.1%)	120 (77.9%)
GGG1	92 (30.5%)	45 (40.2%)	41 (26.6%)
≥ GGG2	136 (45.0%)	48 (42.9%)	79 (51.3%)

^*^ 36 patients received a previous negative biopsy. IQR: interquartile range

**Figure 1:**
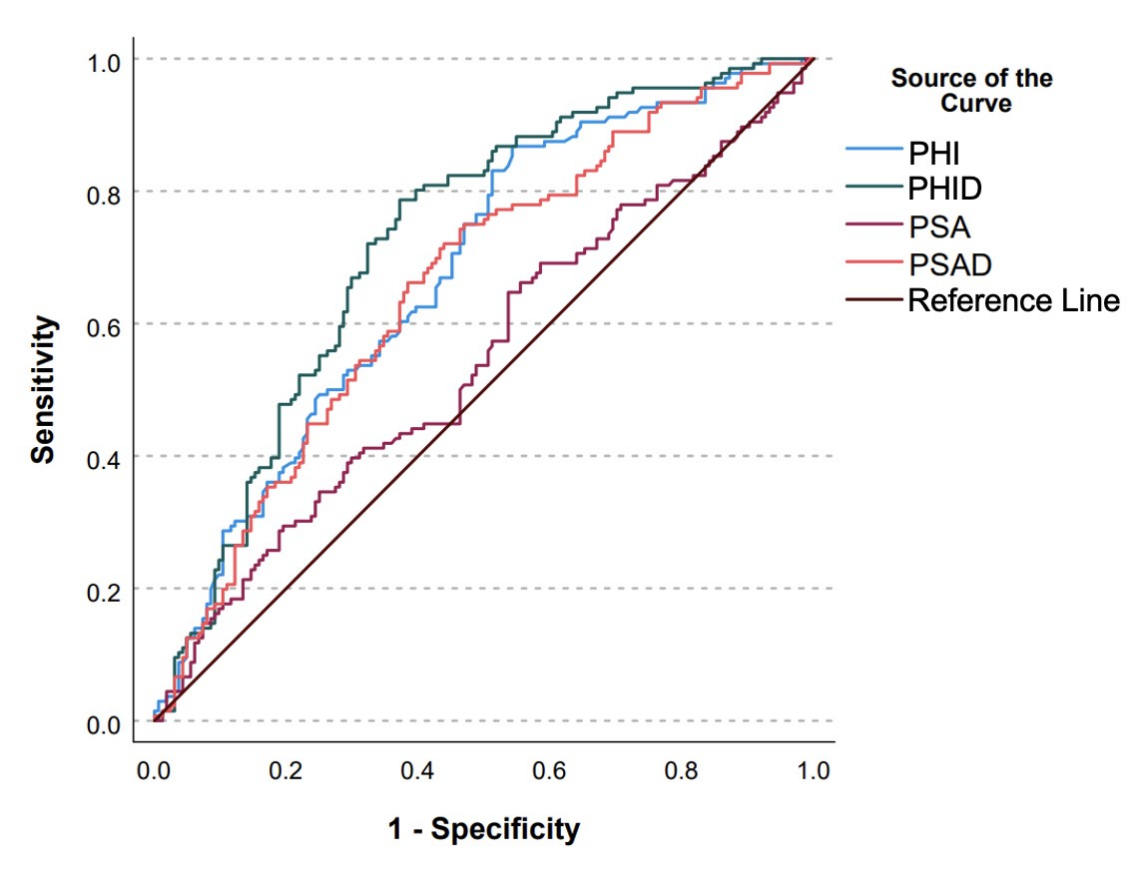
ROC curves for PHI, PHID, PSA, and PSAD. PHI: prostate health index; PHID: prostate health index density; PSA: prostate specific-antigen; PSAD: prostate specific-antigen density

PHI’s sensitivity for detecting PCa with GGG2 or higher at a PHI value above 27 and 36 was 97.0% and 90.4%, respectively. On univariate analysis, PHI values greater than 27 (47.8% vs. 16.0%, *P* = 0.002) and 36 (52.8% vs. 19.1%, *P* < 0.001) were associated with increased detection of ≥ GGG2 PCa. On multivariate analysis, both PHI levels above 27 [odds ratio (OR) 3.9, 95% CI 1.2–12.6, *P* = 0.022] and PHI levels above 36 (OR 4.1, 95% CI 1.9–8.4, *P* < 0.01) remained associated with increased detection of ≥ GGG2 PCa (Table 2, Table 3). In men with PI-RADS ≥ 3, higher PHI was associated with detection csPCa (OR 1.033, 95% CI 1.018–1.049, *P* < 0.001). For patients with PI-RADS lesions < 3, the number of patients who had csPCa was 0% and < 5% for men with a PHI below 27 and 36, respectively. On multivariate analysis, PHID was associated with greater odds of detection of ≥ GGG2 PCa (OR 1.07 per 0.1, 95% CI 1.04–1.3, *P* < 0.001) (Table 4). PHID also had better discriminatory capacity to predict ≥ GGG2 PCa (AUC = 0.725) when compared to PHI (0.677), PSA (0.546), and PSA density (0.658) (Figure 1 & Table 5).

**Table 2:** Multivariate regression for ≥ Gleason grade group 2 prostate cancer

**Variable**	***P*-value**	**OR**	**95% CI for OR**	
			**Lower**	**Upper**
Age	**0.031**	1.040	1.004	1.079
PSA	0.600	1.019	0.949	1.095
PHI > 27	**0.022**	3.925	1.217	12.658
PI-RADS 1–2	Reference			
PI-RADS 3	**0.031**	3.260	1.115	9.533
PI-RADS 4–5	**< 0.001**	11.730	5.464	25.182

OR: odds ratio. Bolded figures are statistically significant (*P* < 0.05)

**Table 3:** Multivariable logistic regression model examining factors associated with detection of Gleason grade group 2 or higher prostate cancer

**Variable**	***P*-value**	**OR**	**95% CI for OR**	
			**Lower**	**Upper**
Age	**0.022**	1.044	1.006	1.084
PSA	0.955	1.002	0.931	1.079
PHI > 36	**< 0.001**	4.102	1.990	8.452
PI-RADS 1–2	Reference			
PI-RADS 3	**0.033**	3.274	1.104	9.712
PI-RADS 4–5	**< 0.001**	11.095	5.125	24.021

OR: odds ratio. Bolded figures are statistically significant (*P* < 0.05)

**Table 4:** Multivariate regression for ≥ Gleason grade 2 prostate cancer

**Variable**	***P*-value**	**OR**	**95% CI for OR**	
			**Lower**	**Upper**
Age	**0.014**	1.052	1.010	1.095
PSA	0.709	1.015	0.939	1.096
PHID	**< 0.001**	1.07	1.039	1.288
PI-RADS 1–2	Reference			
PI-RADS 3	**0.014**	4.206	1.339	13.207
PI-RADS 4–5	**< 0.001**	14.784	6.326	34.553

OR: odds ratio. Bolded figures are statistically significant (*P* < 0.05)

**Table 5:** Area under curve (AUC) values for receiver operating characteristics (ROC) curve

**ROC model**	**Variable**	**AUC**
Overall cohort	PHI	0.677
	> 27	0.549
	> 36	0.620
	PHID	0.725
	> 0.91	0.707
	PSA	0.546
	PSAD	0.658
	> 0.15	0.601
Patients with PI-RADS 1-2 lesions	PHI	
	> 27	0.563
	> 36	0.621
	PHID	
	> 0.91	0.817
	PSAD	
	> 0.15	0.678

PSAD: prostate specific-antigen density

A PHID cutoff of 0.91 was determined based on maximizing the Youden index. At this threshold, the discriminatory ability of PHID to predict ≥ GGG2 PCa was greater than PHI with cutoffs of both 27 and 36 and PSAD cutoff of 0.15 (AUC = 0.707 vs. 0.549 vs. 0.620 vs. 0.601). PHID also improved model fit compared to PHI cutoffs of 27 and 36 and PSAD cutoff of 0.15 (0.435 vs. 0.319 vs. 0.353 vs. 0.349). In our data, if only the PHID cutoff were used, only 44.3% of the original biopsies would have been performed with 35.0% of all of the original biopsies safely able to be avoided (PHID < 0.91 and no ≥ GGG2 PCa) and clinically significant cancers would have been missed in 9.67% of patients who would have been biopsied.

The discriminatory ability of PHID is also increased compared to PHI cutoffs of 27 and 36 and PSAD cutoff of 0.15 (AUC = 0.817 vs. 0.563 vs. 0.621 vs. 0.678) for men with PI-RADS 1–2 lesions. In patients with PI-RADS 1–2 lesions using a PHID cutoff of 0.91, 56.8% of biopsies in patients with PI-RADS 1–2 lesions could have been safely avoided while missing 0 clinically significant cancers. A cutoff of < 0.34 for PHID and cutoff of < 24.5 for PHI for men with PI-RADS 3–5 lesions has a negative predictive value (NPV) of 90% for detection of ≥ GGG2 PCa. A cutoff of > 2.70 for PHID and > 88.75 for PHI in men with PI-RADS 1–2 lesions has a specificity of 90% for detection of ≥ GGG2 PCa. However, subset analysis of men with PI-RADS 1–2 lesions was limited due to lack of statistical power, and thus statistical significance could not be calculated.

Logistic regression was performed across each subset of patients based on biopsy status to evaluate the predictive ability of our new cutoff for PHID of 0.91 vs. PHI cutoff of 27 vs. PHI cutoff of 36. Both PHID > 0.91 (OR 5.6, 95% CI 2.4–12.9, *P* < 0.001) and PHI > 36 (OR 6.1, 95% CI 2.1–17.5, *P* < 0.001) were significant predictors of ≥ GGG2 PCa for biopsy naïve men. Only PHID was able to predict ≥ GGG2 PCa for men with a previous negative biopsy (OR 39.9, 95% CI 2.8–565.4, *P* < 0.001) and men on AS (OR 8.3, 95% CI 2.8–25.1, *P* < 0.001). In patients with a prior negative biopsy, 65.7% of biopsies could have been safely avoided while missing clinically significant cancers in 5.71% of patients who otherwise would have been biopsied. In patients on AS, 35.7% of biopsies could have been safely avoided, while missing clinically significant cancers in 10.71% of patients who would have been biopsied.

## Discussion

We sought to determine if adding PHID cutoff values to MRF-TB, compared to PHI, would augment detection of csPCa. Prostate biopsy is not a benign exam and has been associated with several complications including infection, urinary retention, hematospermia, rectal bleeding, and vasovagal episodes [15], so avoiding unnecessary biopsies is in the best interest of patients.

Though there are variations in clinical practice, in general, men with a PI-RADS 3–5 lesion get a prostate biopsy, while those with a PI-RADS 1–2 may not [16]. With a similar caveat, typically men with a PHI score > 36 typically get biopsies even if PI-RADS is less than 3. Given the high NPV for csPCa of PHI with PI-RADS < 3 lesions, the use of PHID in addition to MRI could more effectively risk stratify men and can reduce the rate of detection of indolent disease and subject men to an unnecessary biopsy. Importantly, these findings are preliminary; futures studies with larger cohorts of PI-RADS 1 and 2 lesions are needed to confirm PHID’s superiority when compared to PHI for risk stratification in this subset.

Our analysis also suggests that PHID is a superior predictor of csPCa when compared to PHI and PSAD due to its higher AUC (0.725 vs. 0.677 vs. 0.658), especially in determining which men with negative MRI should be biopsied [AUC = 0.817 (PHID > 0.91) vs. 0.563 (PHI > 27) vs. 0.621 (PHI > 36) vs. 0.678 (PSAD > 0.15)]. These findings are especially valuable given that PSAD is a valuable adjunctive test to confirm the avoidance of unnecessary prostate biopsy [13, 14]. We have also identified thresholds for both PHI (< 24.5) and PHID (< 0.34) for which men with high-risk MRIs may be able to avoid biopsy with high NPVs. The discriminatory ability of PHID was preserved also across each patient subgroup when stratified by biopsy status. Therefore, we advocate for the use of MRI-determined PHID to evaluate for need for prostate biopsy.

Our results build on prior studies of PHID as a tool for prostate biopsy selection. Importantly, our study is one of the first to evaluate the combination of PHI, PHID, and PI-RADS v2 score for the risk stratification of csPCA in patients in a cohort of over 300 MRF-TB patients. In two studies of cohorts of 241 and 118 men, where csPCa was defined as GGG ≥ 2 or GGG1 detected in more than 2 cores or greater than 50% of any single core, PHID had a high AUC for determining csPCa: 0.780 [11] and 0.84 [10], respectively. In the latter study, PHID, compared to PSA and other PSA-related variables, had the highest discriminative ability for csPCa [9]. Other studies have found no significant benefit of PHID over PHI in identifying csPCa. Stephan et al. [17] found with their cohort of 1,057 men that even though PHID provided a significantly higher AUC than PHI (0.835 vs. 0.801, *P* = 0.0013), there was no significant difference in the detection of csPCa, or Gleason score ≥ 7 PCa (AUC for PHI vs. PHID: 0.74 vs. 0.736, *P* = 0.77). Importantly, these studies did not add PI-RADs v2 for risk stratification. We believe our study also adds to the existing literature evaluating the discriminatory ability of the combination of PHI and MRI imaging in the detection of csPCa. In a cohort of 164 men, a PHI cut-off of 27 had an NPV rate of 87.5% in predicting csPCa in men with PI-RADs 4–5 lesions. However, for this group, the number of patients with PI-RADS 3 lesions was too small to define PHI cutoff [18]. In a cohort of 102 patients, the AUC combining PHI with mpMRI to predict csPCa (Gleason ≥ 7) was greater than that of either alone (PHI: 0.873 vs. 0.735, *P* = 0.002) (mpMRI: 0.873 vs. 0.830, *P* = 0.035) [19]. However, this study did not look at PHID.

There are limitations to this study that should be considered. Gleason grading was based solely on core needle biopsy results and did not include assessments from radical prostatectomy specimens, therefore full pathologic staging cannot be assumed. Our cohort does not represent the entire population of those who underwent screening, but instead a population at higher risk for csPCa, and in particular, those who underwent PHI testing. Our population also represents patients treated at a high-volume academic center with access to MRF-TB which may not be available at other smaller centers. Our population has significant heterogeneity in biopsy status. Despite this, the ability of PHID to predict detection of csPCa was preserved across each patient group. However, given the low cohort size, subset analysis of patients with PI-RADS 1 and 2 patients was not powered enough to reach statistically significant results. As such our findings of PHID’s improved risk stratification for biopsy amoung PI-RADS 1 and 2 patients warrants validation with larger studies and clinical trials.

In conclusion, we support the utilization of PHID to assess the need for prostate biopsy. In our institutional cohort, we found that a PHID cutoff of 0.91 appropriately selects which patients need to undergo biopsy and that PHID showed enhanced predictive capability for detecting ≥ GGG2 PCa compared to widely accepted cutoffs for PHI and PSAD, and potentially among patients with PI-RADS 1–2 lesions. We also found PHI cutoffs of > 27 and > 36 appropriately select which patients need to undergo prostate biopsy, consistent with existing literature.

## Abbreviations

AS: active surveillance
csPCa: clinically significant prostate cancer
GGG: Gleason grade group
IQR: interquartile range
mpMRI: multiparametric magnetic resonance imaging
MRF-TB: magnetic resonance imaging ultrasound fusion-targeted biopsy
MRI: magnetic resonance imaging
NPV: negative predictive value
OR: odds ratio
PCa: prostate cancer
PHI: prostate health index
PHID: prostate health index density
PI-RADS: Prostate Imaging Reporting and Data System
PSA: prostate specific-antigen
PSAD: prostate specific-antigen density
ROC: receiver operating characteristic

## Declarations

### Availability of data and materials

The raw data supporting the conclusions of this manuscript will be made available by the authors, without undue reservation, to any qualified researcher.

### Copyright

© The Author(s) 2024.
